# Mapping UK Pain Datasets to the OMOP Common Data Model: Lessons Learned

**DOI:** 10.23889/ijpds.v9i5.2613

**Published:** 2024-09-18

**Authors:** Gordon Milligan, Erum Masood, Phil Quinlan, Sam Cox, Tom Giles, Armando Mendez Villalon, Christian Cole

**Affiliations:** 1 University of Dundee; 2 University of Nottingham

## Objectives

Chronic Pain is defined as pain lasting for three months or longer and a report by the British Pain Society in 2016 found that 28 million adults live with this condition. Chronic pain research is problematic as it is poorly represented in health data and often in silos. Alleviate is the Advanced Pain Discovery Platform (APDP) Data Hub which is removing barriers to access to data.

## Approach

The aim for Alleviate is to transform pain data sets within the UK to be Findable, Accessible, Interoperable and Reusable (FAIR) and be more discoverable for researchers via Health Data Research UK’s Cohort Discovery Tool. The tool enables identification of potential cohorts in real-time from datasets Alleviate provides. The onboarding requires datasets to be transformed to the open and widely used Observational Medical Outcomes Partnership (OMOP) Common Data Model (CDM).

## Results

We collaboratively developed CaRROT tools and used our mapping expertise to transform datasets to OMOP-CDM. The tools have improved efficient mapping of clinical and research data, covering ~10 million individuals’ records. Subsequently we learned valuable lessons on how to accurately and consistently map across data sets to support federated discovery. We also identified a lack of standard vocabulary representations for pain specific data.

## Conclusions

Mapping data to OMOP is not straightforward. Attention to detail is required to ensure that consistent mapping decisions are made to maximise data reusability and interoperability.

## Implications

In sharing the lessons learned of mapping datasets to OMOP we aim to help improve interoperability of disparate data sources.

